# *Mycoplasma ovipneumoniae* in Wildlife Species beyond Subfamily *Caprinae*

**DOI:** 10.3201/eid2412.180632

**Published:** 2018-12

**Authors:** Margaret A. Highland, David R. Herndon, Scott C. Bender, Lisa Hansen, Robert F. Gerlach, Kimberlee B. Beckmen

**Affiliations:** Washington Animal Disease Diagnostic Laboratory, Pullman, Washington, USA (M.A. Highland);; US Department of Agriculture, Pullman (M.A. Highland, D.R. Herndon);; Navajo Technical University, Crownpoint, New Mexico, USA (S.C. Bender);; Barron Veterinary Clinic, Barron, Wisconsin, USA (L. Hansen);; Alaska Department of Environmental Conservation, Anchorage, Alaska, USA (R.F. Gerlach);; Alaska Department of Fish and Game, Fairbanks, Alaska (K.B. Beckmen)

**Keywords:** Mycoplasma ovipneumoniae, polymerase chain reaction, ribosomal RNA 16S, host specificity, pneumonia, bacteria, reindeer, deer, bison, sheep, goats, zoonoses, respiratory infections, United States

## Abstract

Elucidating the emergence of *Mycoplasma ovipneumoniae*–associated respiratory disease in ruminants requires identification of the pathogen host range. This bacterium was thought to be host restricted to subfamily *Caprinae*, but we describe its identification in healthy moose, caribou, and mule deer and diseased mule and white-tailed deer, all species in subfamily *Capreolinae*.

*Mycoplasma ovipneumoniae* was identified in Queensland, Australia, in 1972 as an infectious agent associated with pneumonia in domestic sheep (*Ovis aries*) (*1*). Since then, it has most frequently been identified in healthy and diseased domestic sheep, domestic goats (*Capra aegagrus hircus*), and bighorn sheep (*Ovis canadensis*). Although *M. ovipneumoniae* was identified in respiratory disease outbreaks in bighorn sheep as early as 1980 (*2*), the past decade has brought it under scrutiny because of evidence supporting its association with bighorn sheep pneumonia in western North America (*3*). Because most reports have described this bacterium in sheep and goats, and fewer in muskoxen (*Ovibos moschatus*) (*4*), some have concluded that *M*. *ovipneumoniae* is specific to the subfamily *Caprinae* (*5*) or has a host range limited to *Caprinae* (*6*), despite publications describing *M. ovipneumoniae* in non-*Caprinae* species, including Beira antelope (*Dorcatragus megalotis*) with respiratory disease in Qatar (*7*) and in 9 cattle (*Bos taurus*) in Colorado, USA (*8*). Unfortunately, description of the method(s) used to identify *M. ovipneumoniae* in those reports was limited to stating the use of PCR with no supporting sequence data.

In general, definitive claims of host range restrictions are absent from mycoplasma literature, because “assumptions about restricted host range of mycoplasmas, based on the host from which they were first or frequently isolated, are usually made in the context of nearly complete absence of representative sampling of the vast majority of potential vertebrate hosts” (*9*). In addition to insufficient sampling of potential hosts, the fastidious and variably culturable nature of *M. ovipneumoniae* often requires molecular techniques for identification. We used molecular techniques to analyze multiple species from the subfamily *Capreolinae* for the presence of *M. ovipneumoniae*.

During July 2017–January 2018, the US Department of Agriculture Agricultural Research Service in Pullman, WA, USA, received nasal swab samples from 230 moose (*Alces alces*) and 243 caribou (*Rangifer tarandus*) from Alaska and 5 mule deer (*Odocoileus hemionus*) from Arizona (Technical Appendix). Also received in February 2018 was an isolate of *M. ovipneumoniae* that had been cultured by Newport Laboratories (Worthington, MN, USA) from lung tissue from a white-tailed deer (*Odocoileus virginianus*) that died during a pneumonia outbreak at a captive facility in the upper Midwest region of the United States in 2016. We extracted DNA from swab samples and from the white-tailed deer isolate, performed PCR using a modified published PCR method (*10*) to amplify part of the 16S rRNA gene, and sequenced amplicons of the correct size (Technical Appendix). Forward and reverse sequences were merged, manually inspected for errors, and trimmed to 290 bp using Sequencher 5.2.2 (Gene Codes, Ann Arbor, MI, USA) corresponding to a 103–392-bp region of the 16S rRNA gene of type strain Y98 obtained from American Type Culture Collection (ATCC; Manassas, VA, USA). Sequences were blastn queried (https://www.ncbi.nlm.nih.gov/BLAST/) and identified as *M. ovipneumoniae* at 100% coverage and ≥97% identity to Y98 (GenBank accession no. NR_025989.1). The analyzed region represents the most divergent region of the 16S rRNA gene among strains of *M. ovipneumoniae* and between *Mycoplasma* spp. of the highest percentage identity to *M. ovipneumoniae* (Technical Appendix).

We detected *M. ovipneumoniae* in 6 moose (2.6%), including 3 from 2 captive facilities and 3 free-ranging; 5 free-ranging caribou (2.1%); and 2 of 5 mule deer, 1 of which was coughing and had nasal discharge at the time of sample collection. The identity of the lung isolate, cultured from the white-tailed deer that had died from pneumonia, was confirmed as *M. ovipneumoniae*. For sequence comparison, we generated a percent identity matrix with the *M. ovipneumoniae* sequences from the *Capreolinae* species, nasal swabs collected from 2 healthy *M. ovipneumoniae*–positive wild sheep (Technical Appendix), Y98, and bacteria of the closest identity to Y98 and sequences generated in this study based on blastn queries (*M. dispar*, *M. hyopneumoniae*, and *M. flocculare*) (Technical Appendix). The percent identity matrix revealed 2 groupings of *M*. *ovipneumoniae* and illustrates the divergence from the other *Mycoplasma* spp. of closest identity to *M. ovipneumoniae*. Sample sequences have been submitted to GenBank (Technical Appendix).

This report describes *M. ovipneumoniae* carriage in multiple members of the subfamily *Capreolinae* (moose, caribou, and mule deer), and emergence of *M. ovipneumoniae*–associated respiratory disease in deer. These findings are of importance to epidemiologic investigations because current dogma regarding host specificity may dissuade laboratories from pursuing identification of this fastidious bacterium in hosts beyond the subfamily *Caprinae*. Improved diagnostic methods to increase detection sensitivity are warranted based on information provided in this report (Technical Appendix). Full-length genome sequencing and phylogenetic analysis of *M. ovipneumoniae* isolates are necessary next steps in inferring evolutionary relationships and origin of this bacterium in identified host species.

Technical Appendix Discussion of materials and methods used in the study of *Mycoplasma ovipneumoniae* in moose, caribou, and deer.
