# Supplementary material for: Mycoplasma ovipneumoniae in Wildlife Species beyond Subfamily Caprinae
Source: Emerg Infect Dis. 2018 Dec;24(12):2384–6. doi: 10.3201/eid2412.180632 (PMC6256407; doi:10.3201/eid2412.180632)
Supplement: Technical Appendix — Discussion of materials and methods used in the study of Mycoplasma ovipneumoniae in moose, caribou, and deer. [file 18-0632-Techapp-s1.pdf]

Article DOI: https://doi.org/10.3201/eid2412.180632

Mycoplasma ovipneumoniae in Wildlife Species beyond Subfamily *Caprinae*

Technical Appendix

Materials and Methods

Animals (Study Samples and PCR Controls)

We collected nasal swab samples from moose and caribou during October 2013–December 2017, and from mule deer in January 2018, during routine health monitoring captures. Nasal swabs were placed into universal viral transport media (UVT; Becton Dickinson, Franklin Lakes, NJ, USA), sterile phosphate buffered saline, or a dry transport cylinder. Samples collected during 2017–2018 were kept cool and shipped on ice packs within 48 hours of collection, whereas samples collected before 2017 were maintained at −70°C following collection and shipped on dry ice. Nasal swab samples from *M. ovipneumoniae*-positive healthy wild sheep, including a Dall’s sheep in Alaska, USA and a bighorn sheep in Colorado, USA, were collected during health monitoring captures, placed in UVT, and sent on ice packs following collection in November 2017 and March 2017, respectively. Sequence data from these *M. ovipneumoniae*-positive wild sheep are used for sequence identity comparison, representing species that have previously been reported as *M. ovipneumoniae* hosts.

PCR-positive control samples included DNA extracted from a nasal swab collected from a *M. ovipneumoniae*-positive domestic sheep in February 2016 in Idaho, USA, and from American Type Culture Collection type strain Y98 culture (domestic sheep origin). Negative control samples included DNA extractions, performed alongside test samples, on PCR-grade water, and/or *M. ovipneumoniae*-negative domestic sheep. Negative control sheep were raised and maintained under specific pathogen-free conditions (*1*) and confirmed *M. ovipneumoniae*-free by serology (Washington Animal Disease Diagnostic Laboratory, Pullman, WA) and repeat nasal swab sample analysis, as described in this study.

DNA Isolation, PCR, Sequencing, Sequence Analysis

We extracted DNA from swab samples and from the white-tailed deer isolate, and performed PCR using commercial kits (QIAamp DNA Mini Kit and QIAGEN Multiplex PCR Kit; QIAGEN, Germantown, MD, USA). We performed DNA extractions using the manufacturer's protocol titled "DNA Purification from Buccal Swabs (Spin Protocol)" (QIAamp DNA Mini and Blood Mini Handbook, 05/2016) with the following modifications: 1 mL of digest was applied to the spin column by placing 500 μL of sample digest onto the column, centrifuged, then repeated; samples were eluted with 100 μL of elution buffer provided in the kit. For samples arriving in 3 mL UVT media, 400 μL of media, in place of the initial PBS step, was used to perform the same extraction procedure. DNA eluent from dry swab samples or swabs arriving in small volumes of PBS were diluted 10-fold before performing PCR, to dilute potential inhibitor carry-through. Published primers (*2*) that amplify part of the 16S rRNA gene, base range 66–426 of type strain NCTC 10151 [Y98] (ATCC 29419; Manassas, VA, USA), were used at a 1 μM combined concentration in 20 μL reactions containing 2 μL of sample DNA and 10 μL of Multiplex PCR Master Mix (QIAGEN). PCR cycler conditions were as follows: 15 min denaturation, followed by 40 cycles, each 30 s, of 95°C denaturation, 58°C annealing, and 72°C extension, then a final 10 min extension, and 4°C hold. We performed gel electrophoresis on an aliquot of each PCR reaction, including negative and positive controls. Each reaction having a visualized amplicon of correct size was Sanger sequenced (Eurofins Genomics, Louisville, KY, USA). Sequences generated in this study were GenBank accessioned (Technical Appendix Table).

PCR Method Detection Limit

The method detection limit (MDL) for DNA extracted from Y98 culture was determined to be 10^1 templates per 2 μL. MDL for biologic samples collected and submitted as dry nasal swabs was determined to be 10^4 (10^4 per 1.2 mL digestion solution, which equates to 14 copies per 2 μL of the final 10-fold diluted eluent used in PCR reaction). This was determined by spiking *M. ovipneumoniae* negative domestic goat nasal swab samples with log dilutions of extracted DNA from Y98 culture before performing DNA extraction on the swab sample. The MDL for 10-fold diluted extracted DNA from negative swab samples spiked with Y98 extracted DNA was determined to be 10^2 per 2 μL. This supports high DNA extraction efficiency and low

PCR inhibitors from the biologic sample with ≤1 log fold decrease in MDL between pure culture and biologic samples.

Divergence Analysis

To illustrate the 290 bp region of the 16S rRNA gene analyzed in this study, in positional relation to variable regions of the 16S rRNA gene of *M. ovipneumoniae* and bacteria of the closest identity, a conservation plot was generated by graphing conservation of a multiple sequence alignment using the default settings in CLC Genomics Workbench (Redwood City, CA, USA) (Technical Appendix Figure 1). Bacteria of closest identity were *Mycoplasma* spp. and are referenced in the Technical Appendix Table.

Percent Identity Matrix

To compare sequences described in this study to the Y98 *M. ovipneumoniae* type strain and bacteria of the closest identity to Y98, a multiple sequence alignment was performed followed by a pairwise sequence comparison using default setting in CLC Genomics Workbench (QIAGEN, Redwood City, CA, USA); we then applied conditional formatting in Microsoft Excel (Redmond, WA, USA) (Technical Appendix Figure 2).

References

1. Highland MA, Berglund AK, Knowles DP. Total IgG in ewe sera and colostrum and serum IgG kinetics in lambs following colostrum ingestion are similar in domestic sheep and bighorn sheep (*Ovis aries* and *Ovis canadensis*). Sheep Goat Res J. 2017;32:36–42.
2. McAuliffe L, Hatchell FM, Ayling RD, King AI, Nicholas RA. Detection of *Mycoplasma ovipneumoniae* in *Pasteurella*-vaccinated sheep flocks with respiratory disease in England. Vet Rec. 2003;153:687–8. PubMed http://dx.doi.org/10.1136/vr.153.22.687

Technical Appendix Table. GenBank accessioned partial 16S rRNA *Mycoplasma ovipneumoniae* sequences and reference strains

Species	Animal identification	GenBank accession no.
Alaskan moose (*Alces alces*)	13_12–20–2017	MH133232
	30_01–24–2018	MH133235
	25_01–25–2018	MH133233
	41_01–25–2018	MH133236
	28_02–09–2018	MH133234
	86_01–24–2018	MH133237
Caribou (*Rangifer tarandus*)	76_02–14–2018	MH133229
	94_02–14–2018	MH133230
	51_12–20–2017	MH133228
	24_12–29–2017	MH133227
	23_01–09–2018	MH133226
Mule deer (*Odocoileus hemionus*)	93_12–04–2017	MH133239
	90_12–04–2018	MH133238
White-tailed deer (*Odocoileus virginianus*)	01_03–16–2018	MH133240
Bighorn sheep (*Ovis canadensis*)	62_04–07–2016	MH133224
Dall's sheep (*Ovis dalli*)	77_12–04–2017	MH133231
Reference strains	Strain designation	
Mycoplasma ovipneumoniae	NCTC 10151 [Y98]	NZ_JOTL01000000
Mycoplasma dispar	NCTC 10125	NZ_CP007229
Mycoplasma flocculare	[Ms42, NCTC 10143]	CP007585
Mycoplasma hyopneumoniae	NCTC 10110; J	NC_007295

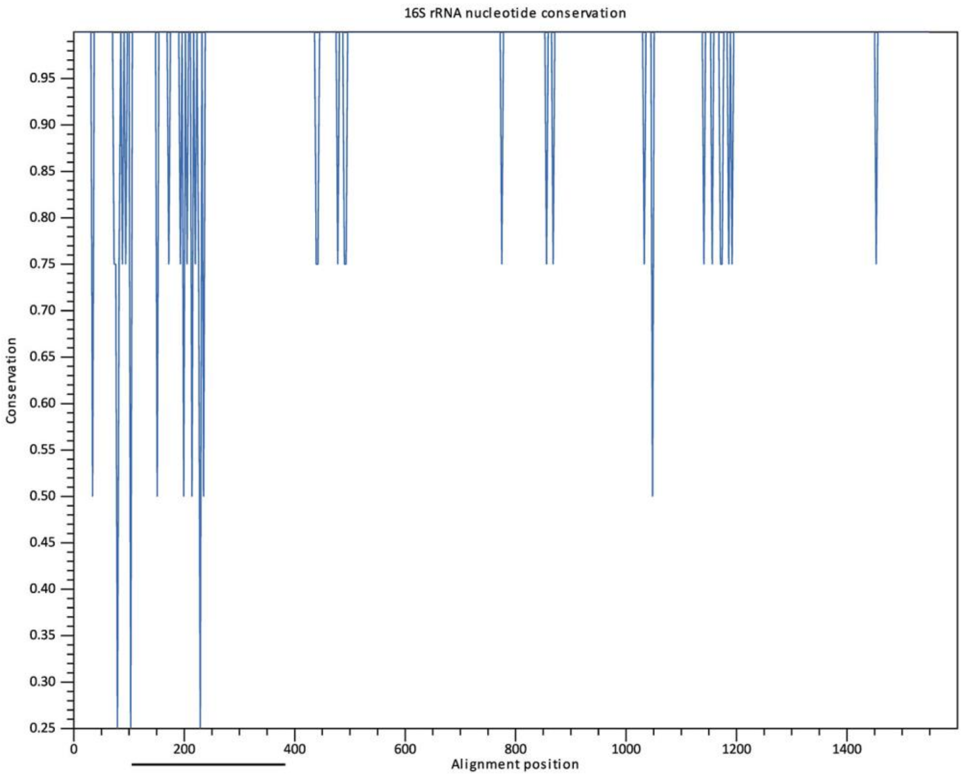

Technical Appendix Figure 1. Full-length 16S rRNA gene multiple sequence alignment conservation graph of *M. ovipneumoniae* type strain Y98 and the three mycoplasmas of the highest percent identity to Y98: *M. dispar*, *M. flocculare*, and *M. hyopneumoniae*. Black line illustrates the 290 bp region used in this study to identify *M. ovipneumoniae* in samples.

		1	2	3	4	5	6	7	8	9	10	11	12	13	14	15	16	17	18	19	20
Mycoplasma ovipneumoniae	1		0	1	1	1	1	1	2	7	7	7	7	7	7	7	7	9	15	17	20
Moose 25 01-25-18	2	100		1	1	1	1	1	2	7	7	7	7	7	7	7	7	9	15	17	20
Moose 30 01-24-18	3	100	100		2	0	0	2	1	7	7	7	7	7	7	7	7	9	14	16	19
Moose 41 01-25-18	4	100	100	99.31		2	2	2	3	8	8	8	8	8	8	8	8	10	16	18	21
Mule deer 90 02-04-17	5	100	100	100	99.31		0	2	1	7	7	7	7	7	7	7	7	9	14	16	19
Mule deer 93 12-04-17	6	100	100	100	99.31	100		2	1	7	7	7	7	7	7	7	7	9	14	16	19
Caribou 76 02-14-18	7	100	100	99.31	99.31	99.31	99.31		3	8	8	8	8	8	8	8	8	10	15	17	20
Moose 13 12-20-17	8	99.31	99.31	100	98.97	100	100	98.97		8	8	8	8	8	8	8	8	10	15	17	20
Bighorn sheep 62 04-07-17	9	97.59	97.59	97.59	97.24	97.59	97.59	97.24	97.24		0	0	0	0	0	0	0	2	15	19	18
Caribou 23 01-09-18	10	97.59	97.59	97.59	97.24	97.59	97.59	97.24	97.24	100		0	0	0	0	0	0	2	15	19	18
Caribou 24 12-29-17	11	97.59	97.59	97.59	97.24	97.59	97.59	97.24	97.24	100	100		0	0	0	0	0	2	15	19	18
Caribou 51 12-20-17	12	97.59	97.59	97.59	97.24	97.59	97.59	97.24	97.24	100	100	100		0	0	0	0	2	15	19	18
Caribou 94 02-14-18	13	97.59	97.59	97.59	97.24	97.59	97.59	97.24	97.24	100	100	100	100		0	0	0	2	15	19	18
Dall's sheep 77 12-04-17	14	97.59	97.59	97.59	97.24	97.59	97.59	97.24	97.24	100	100	100	100	100		0	0	2	15	19	18
Moose 86 01-24-18	15	97.59	97.59	97.59	97.24	97.59	97.59	97.24	97.24	100	100	100	100	100	100		0	2	15	19	18
White-tailed deer NL 06-22-16	16	97.59	97.59	97.59	97.24	97.59	97.59	97.24	97.24	100	100	100	100	100	100	100		2	15	19	18
Moose 28 02-09-18	17	96.90	96.90	96.90	96.55	96.90	96.90	96.55	96.55	99.31	99.31	99.31	99.31	99.31	99.31	99.31	99.31		17	19	18
Mycoplasma dispar	18	94.83	94.83	95.17	94.48	95.17	95.17	94.83	94.83	94.83	94.83	94.83	94.83	94.83	94.83	94.83	94.83	94.14		15	13
Mycoplasma hyopneumoniae	19	94.14	94.14	94.48	93.79	94.48	94.48	94.14	94.14	93.45	93.45	93.45	93.45	93.45	93.45	93.45	93.45	93.45	94.83		15
Mycoplasma flocculare	20	93.13	93.13	93.47	92.78	93.47	93.47	93.13	93.13	93.81	93.81	93.81	93.81	93.81	93.81	93.81	93.81	93.81	95.53	94.85	

Technical Appendix Figure 2. Percent identity matrix illustrating pairwise sequence comparison of percent identity (lower left) and number of nucleotide changes (upper right) for the analyzed 290 bp of the 16S rRNA gene (range 103–392 of type strain Y98 *Mycoplasma ovipneumoniae*) from samples, type strain Y98 *M. ovipneumoniae*, and the next 3 closest blastn (https://www.ncbi.nlm.nih.gov/BLAST/) sequence query matches: *M. dispar*, *M, hyopneumoniae*, and *M. flocculare*. Percent identity and nucleotide change values grouped as follows: 98%–100% (≤3 changes), 96%–98% (4–12 changes) and 90%–96% (≥12 changes).
